# Sentinel events in pediatric hepatology: A pilot simulation curriculum

**DOI:** 10.1002/jpr3.70133

**Published:** 2026-01-07

**Authors:** Anne Lyon, Alan Leichtner, Donna Luff, Scott Elisofon, Andrew Wehrman, Katherine Sweeny, Janet MacDonald, Christine K. Lee

**Affiliations:** ^1^ Division of Pediatric Gastroenterology, Hepatology, and Nutrition Boston Children's Hospital Boston Massachusetts USA; ^2^ Department of Anesthesia Immersive Design Systems, Harvard Medical School Boston Massachusetts USA

**Keywords:** fellowship, medical education, pediatric gastroenterology

## Abstract

**Objectives:**

Sentinel hepatology events require robust prior experience to accurately diagnose and manage. Given the rarity of complex pediatric liver disease, gastroenterology (GI) fellows do not uniformly get exposure to these patients. We developed a simulation curriculum to ensure exposure to and improve confidence in diagnosis and management of these patients.

**Methods:**

Using needs assessment data, a simulation experience was developed, including two clinical scenarios, each mimicking several days of case evolution. Learning objectives included effective communication of diagnostic and management plans to medical teams and families. Targeted didactics were completed between phases. At the conclusion of the experience, fellows scored their confidence levels across learning objectives compared to a retrospective baseline. To assess longitudinal confidence, two similar online clinical cases were disseminated 5 months later with repeat confidence scoring.

**Results:**

Six of eight eligible fellows participated in the simulation experience. All participants reported that the curriculum was meaningful and decreased their anxiety caring for these patients. The collaborative structure fostered psychological safety and mitigated cognitive load. There was a statistically significant increase in self‐assessed confidence for all learning objectives after the simulation.

**Conclusion:**

In the current training environment, it is difficult to attain autonomy in managing complex pediatric liver disease. This simulation is the first of its kind in pediatric GI to address diagnosis and management of complex chronic liver disease. The pilot was feasible, impactful, and could help standardize fellow training regardless of program size or transplant center designation, thereby impacting the care of children with liver disease.

## Abstract (highlights)

**What is Known:**

- The American Board of Pediatrics (per Pediatric Gastroenterology board content outline) expects all trainees to be proficient in the recognition, triage, diagnosis, and initial management of complex pediatric liver disease.
- There is no known simulation curriculum focused on diagnosis and management of chronic disease across a hospitalization.

**What is New:**

- Simulation is a feasible modality to teach and reinforce the diagnosis and management of pediatric complex liver disease.
- Simulation can successfully be used by medical subspecialties to bolster trainee education and can be adapted to focus on the care of patients with chronic disease over longer time periods.

## 1 INTRODUCTION

Successful medical training relies on adequate and repeated exposure to a variety of diseases, with the old adage “practice makes perfect” ringing true. In fact, the volume‐outcome relationship, first described by Luft in 1979, highlights that higher hospital volume often leads to improved clinical outcomes.^1^, ^2^, ^3^, ^4^ Primarily studied in the surgical fields, this relationship is more difficult to apply to pediatric medicine. The very nature of pediatrics is such that most children are well, complex pathophysiology is seen infrequently and is often routed to quaternary care centers. In pediatric hepatology, patients on average have high complexity and acuity, and the sickest of those patients often require care in liver transplant centers. This further isolates the exposure and expertise to a smaller percentage of pediatric gastroenterology (GI) trainees and faculty. While some fellows train at these high‐volume, high‐complexity liver transplant centers, the majority do not. Even within pediatric GI fellowship programs at these centers, an individual trainee's exposure to cases may be variable and insufficient to ensure competency. Still, the American Board of Pediatrics (ABP) expects all trainees to be proficient in the recognition, triage, diagnosis, and initial management of complex pediatric liver disease. To offer sufficient exposure and consistency of experience across and within pediatric GI fellowships, innovative educational curricula are needed.

Until now, simulation‐based medical education during fellowship has been overwhelmingly focused on procedural skill acquisition, resuscitation, and team‐based communication, primarily in the surgical, critical care, and emergency medicine fields.^5^, ^6^, ^7^, ^8^, ^9^ Within GI, it has been used mainly for endoscopy skills training in adult GI.^10^, ^11^, ^12^ Mannarino et al. outlined the development of a simulation‐based pediatric critical care curriculum explicitly focused on teaching fellows complex pathophysiology in critically ill patients^13^; however, simulation is rarely used for this purpose. In pediatric GI fellowship training, Solomon et al. have reported on the feasibility of a novel observed structured clinical examination (OSCE) program tailored to the pediatric GI experience and focused on assessment of four core Accreditation Council for Graduate Medical Education (ACGME) competencies—patient care, medical knowledge, interpersonal skills/communication, and professionalism.^14^

To date, there are no published studies describing simulation aimed at diagnosis and management of chronic liver disease over a hospitalization. A curriculum of this kind could augment real patient encounters and leave trainees with robust clinical experiences caring for chronic liver disease to draw on as practicing clinicians. Simulation could, in part, be the solution to the clinical volume dilemma that is seen in subspecialty pediatrics.

We hypothesized that the pediatric GI fellows at Boston Children's Hospital (BCH), a quaternary referral liver transplant center, would report their experience and active management during sentinel pediatric hepatology events as variable and their confidence in the ability to manage future patient events as low. Given this educational gap, our aim was to utilize simulation to teach and reinforce complex pathophysiology of hepatic disorders as part of real‐time clinical decision‐making practice, and we hypothesized that a simulation experience with targeted, interspersed didactics would improve trainee confidence in caring for patients with complex liver disease. Further, we aimed to test the durability of this learning experience with additional online case‐based assessment.

## 2 METHODS

### 2.1 Ethics statement

This study was determined to be exempt from review by the BCH Investigational Review Board. As a framework for the creation of our simulation, we utilized Kern's Six‐Step Approach to Curriculum Development.^15^

### 2.2 Targeted needs assessment

In Fall 2021, a needs assessment was conducted to determine the current status of hepatology training at BCH, focusing both on exposure to a variety of liver pathology as well as opportunity for and confidence in primary management of these patients. The specific liver diseases included were in alignment with the ABP Content Outline for Pediatric Gastroenterology, a blueprint defining the categories contained in the subspecialty certification exam and deemed important for practicing GI physicians.^16^ The needs assessment survey was sent via SurveyMonkey® to all current BCH GI fellows (*n* = 14) as well as the 2021 BCH GI fellowship graduates (*n* = 4). The initial survey dissemination occurred in November 2021, with one reminder sent. Of note, the timing of the needs assessment was such that all first‐year fellows had already completed at least one block of inpatient hepatology.

### 2.3 Case design and development

Two clinical cases were designed based on the needs assessment results. The cases were developed using deidentified data from real patient encounters and had associated learning objectives formulated using the ABP Pediatric GI Certifying Exam Content Outline to ensure alignment with what a pediatric gastroenterologist is expected to know upon completion of training. Each case highlighted the inpatient evolution of a clinical problem over several days. The cases were reviewed by content experts (four pediatric hepatology attending physicians, including the primary investigator) for accuracy, realism, and appropriate level of difficulty. Simulation educators and technical operations specialists at BCH Immersive Design Systems reviewed manikin and technical needs for the cases and collaborated on design and delivery of the simulation. The final simulation approach was consistent with a Zone 2 simulation design. including an emphasis on contextualized skills building, scenario realism, and the use of embedded actors to enhance the experience.^17^

The clinical cases are summarized as follows, with detailed learning objectives in Table 1.

**Table 1:** Clinical case learning objectives.

Case 1—Acute variceal bleed	Learning objectives
Phase 1	1. Describe the initial management of an acute GI bleed, including potential interventions. 2. Generate a robust differential diagnosis for an acute upper GI bleed in a previously healthy patient.
Phase 2	3. Anticipate potential findings on EGD evaluation of an upper GI bleed as well as equipment that may need to be utilized. 4. Describe specialized endoscopic management of an acute variceal bleed, including sclerotherapy and variceal band ligation. 5. Demonstrate setting up the variceal banding apparatus on the endoscope.
Phase 3	6. Describe specialized medical management of an acute variceal bleed including transfusion criteria and use of antibiotics, octreotide.
Phase 4	7. Discuss the possible etiologies of portal vein thrombosis. 8. Describe the long‐term management of portal hypertension and esophageal varices in this patient.

**Case 2—Decompensated cirrhosis**	**Learning objectives**
Phase 1	1. Explain etiology and pathophysiology of hyponatremia and ascites in a cirrhotic patient.
Phase 2	2. Classify the severity, chronicity of hyponatremia. 3. Explain the potential complications of diuretic use and sodium correction in this patient.
Phase 3	4. Determine the need for paracentesis in this patient. 5. Understand the logistics of paracentesis (e.g., how much fluid off, what analysis should be ordered, and possible albumin infusion). 6. Explain the potential complications of paracentesis in this type of patient.
Phase 4	7. Interpret fluid sent from paracentesis. 8. Discuss the longer‐term management of decompensated cirrhosis.

Abbreviations: EGD, esophagogastroduodenoscopy; GI, gastrointestinal.

#### 2.3.1 Case 1: Acute variceal bleeding

This case follows a previously healthy 2‐year‐old patient presenting with hematemesis and melena, who was ultimately found to have esophageal varices and portal hypertension in the setting of a portal vein thrombosis. The case objectives include proficiency in evaluation and management of acute, undifferentiated GI bleeding, variceal bleeding, as well as an understanding of etiology of portal hypertension and portal vein thrombosis.

#### 2.3.2 Case 2: Decompensated cirrhosis

This case follows a 4‐month‐old girl with a late diagnosis of biliary atresia with cirrhosis and ascites presenting with weight gain and severe hyponatremia. The case objectives include recognition and understanding of hyponatremia and ascites in cirrhosis, as well as proficiency in the management of decompensated cirrhosis and possible complications associated with treatments.

### 2.4 Implementation

Two cohorts of senior fellows participated in a 4‐h simulation experience at the BCH simulation center. It was an optional experience that was presented to trainees as formative, not summative in nature.

The simulations took place in August 2022 (*n* = 3) and May 2023 (*n* = 3), when the participants were at the start of the third and end of the second fellowship year, respectively. This cohort of fellows was chosen to minimize participant variability, with BCH fellows completing most, if not all, of their inpatient hepatology service time by the end of their first year.

Each simulation session began with an orientation to the activity and space. This was followed by participation in two distinct patient cases, each lasting 1 h and consisting of four distinct phases. In each phase of the simulation, a team of two fellows actively participated and were tasked with 1–3 learning objectives, interacting with a high‐fidelity manikin and 1–2 participating actors who were pediatric hepatologists (acting as trainees and attendings) as well as a liver program nurse (acting as family member). One fellow passively observed the session inside the room. The fellows rotated roles throughout the experience.

All fellow participants remained in the simulation room throughout the afternoon, while participating actors and faculty observers were stationed in a separate observation room. The simulation director (A. L.) and simulation educator (D. L.) acted as facilitators and directed in‐room simulation changes with a technician in the control room. All simulation activities were filmed with participant consent, and transcripts were obtained.

Fellow teams were provided with written clinical information as well as specific prompts to facilitate interaction with the manikin, clinical decision‐making practice, and engagement with the actors. The actors were provided a copy of the clinical information alongside scenario‐specific learning objectives and guidelines for interaction (“if then” statements) with fellow participants.

A faculty observer utilized a checklist to track participant performance, which included observable behaviors and ideal clinical responses based on the learning objectives. At the conclusion of each phase, the simulation educator (D. L.) led a short debrief for fellow teams to reflect, before the faculty observer offered in‐the‐moment feedback as well as standardized content review prior to moving to the next phase.

After completion of the 4‐h simulation session, an extended debrief was held using the plus/delta method to identify what went well and what could have gone differently for each case, as well as the overall experience.^18^

### 2.5 Evaluation

Fellow participants were provided with a paper‐based survey at the conclusion of the experience, including standardized questions developed by BCH Immersive Design Systems to measure quality, psychological safety, and participant anxiety level, as well as open‐ended feedback questions to elicit areas of strength and possible improvement in the program. The survey also asked each participant to score their confidence level (0 being novice, 100 being expert) across the case learning objectives after having completed the simulation, with a retrospective scoring of their confidence levels prior to the experience. The difference between the baseline and postintervention confidence levels was analyzed using the Wilcoxon signed‐rank test.

### 2.6 Spaced learning—Online experience

Five months after the simulation, fellow participants were presented with an online learning experience consisting of two similar clinical cases created using a Google Forms interface and including clinical questions with free‐text responses. There were no additional didactic components. At the conclusion of these cases, fellow participants were again asked to score their confidence level across the learning objectives after having completed the online experience, as well as retrospectively just prior to the online cases. Of note, confidence in assembly of the variceal banding equipment was not queried after the online case, as there was no method of incorporating manual demonstration into the online modality.

## 3 RESULTS

### 3.1 Needs assessment

Twelve responses to the needs assessment survey were received from 11/14 current GI fellows and 1/4 fellow graduates. All respondents reported that they had cared for patients with neonatal cholestasis and acute hepatitis. Moreover, 83% (10/12) of the respondents had cared for patients in acute liver failure. When asked if they had cared for other complex liver diseases, responses were more variable for acute variceal bleeding (75%, 9/12), refractory ascites (58%, 7/12), and hyponatremia in decompensated cirrhosis (67%, 8/12).

**Figure 1:**
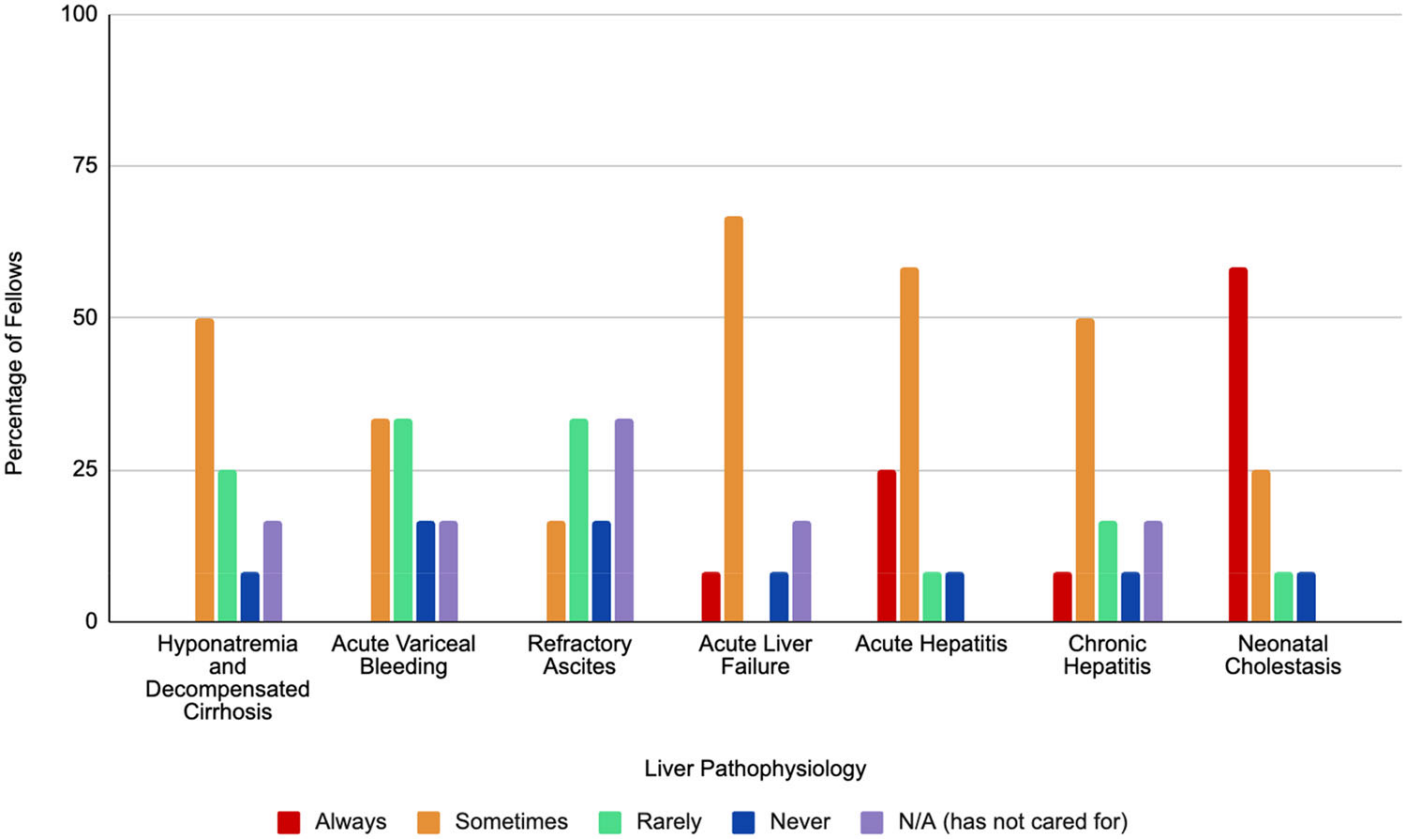
Bar graph depicting frequency of fellows (*n* = 12) as primary communicators in the care of complex hepatology patients. Red bars represent “always,” orange bars represent “sometimes,” green bars represent “rarely,” blue bars represent “never,” purple bars represent “has not cared for.” N/A, not applicable.

When asked about being the leader in clinical care discussions for patients with complex liver disease including acute variceal bleeding, refractory ascites and hyponatremia in decompensated cirrhosis, no respondents felt that they were always the primary physician leading discussions and 60% (6/10), 75% (6/8), and 40% (4/10) of respondents, respectively, felt they were rarely or never the primary physician. These results are summarized in Figure 1.

When citing reasons that respondents were not leading discussions as primary physicians, 89% (8/9) of respondents felt they did not have enough clinical experience or understanding of pathophysiology to do so effectively, and 56% (5/9) of respondents felt they were not given the opportunity.

All respondents felt they could benefit from more practice caring for more complex liver disease and pointed to multiple helpful learning modalities including in‐person simulation (73%, 8/11), online learning experiences (55%, 6/11), OSCE (18%, 2/11), formal lectures (55%, 6/11), and a repository of online resources and guidelines (55%, 6/11).

When analyzed by postgraduate year, there was a trend towards more experience caring for complex liver disease with more time in fellowship. Although only 25%–50% of first‐year fellows had cared for patients with acute liver failure, variceal bleeds, hyponatremia in decompensated cirrhosis, and ascites, all third‐year fellows had encountered these patients. Despite this increase in exposure, the majority (67%–100%) of third‐year fellows felt that they were only sometimes to rarely the primary physician leading discussions for these patients.

### 3.2 Simulation experience

Several themes emerged from qualitative review of the participant surveys and during in‐person feedback on the days of the simulation, including:

1. Collaborative learning environment—Defined as learning that involves peers/pupils working together on activities in a group small enough to ensure everyone participates. Most fellows reported that this was instrumental in the experience.
2. Prompt content review—All fellows reported that immediate clinical review and didactics in between scenarios were an element that significantly supported their learning.
3. Safe, active learning environment—All fellows strongly agreed that the simulation was a safe environment where they were active participants. The simulation curriculum met targeted learning objectives and had meaningful debriefing opportunities.

All fellows indicated that the simulation curriculum was a meaningful experience, which helped them feel less anxious caring for complex liver disease, and recommended integration into the standard fellowship curriculum.

All fellow participants self‐reported increased confidence across all 12 learning objectives after the in‐person simulation experience. Aggregating the six participants' confidence levels for each learning objective, a Wilcoxon signed‐rank test was performed to evaluate the significance of the differences in confidence across the experience. This test indicated a statistically significant and large difference (*p* < 0.05) in median self‐assessed confidence across all 12 learning objectives after the in‐person simulation experience compared to baseline, as outlined in Table 2.

**Table 2:** Difference in median confidence across all learning objectives, pre‐/postsimulation.

Case—Learning objective	Presimulation median confidence (IQR), *n* = 6	Postsimulation median confidence (IQR), *n* = 6	*p* value
1–1	60 (50–80)	88.5 (77–99)	0.031
1–2	50 (50–63)	75 (75–76)	0.035
1–3	15 (0–23)	67.5 (63–75)	0.035
1–4	50 (25–65)	76 (75–80)	0.031
1–5	42.5 (25–50)	76 (75–80)	0.031
2–1	50 (50–62)	75 (75–75)	0.033
2–2	36.5 (25–50)	75 (75–75)	0.035
2–3	50 (50–55)	75 (75–82)	0.034
2–4	47.5 (25–50)	75 (75–82)	0.035
2–5	50 (50–56)	75 (75–82)	0.034
2–6	50 (50–70)	75 (75–82)	0.034
2–7	49 (25–50)	75 (75–75)	0.035

Abbreviation: IQR, interquartile range.

All participants had some degradation in confidence over the intervening 5 months between the initial experience and online cases. There was, however, an increase in self‐assessed confidence after the spaced learning activity in most learning objectives as outlined in Table 3. We were unable to perform any statistical analysis, given less than 100% response rate and minimum numbers needed for the Wilcoxon signed‐rank test to be applied.

**Table 3:** Difference in median confidence across all learning objectives, spaced learning.

Case—Learning objective	Postsimulation median confidence (IQR), *n* = 6	Prespaced learning median confidence (IQR), *n* = 5	Postspaced learning median confidence (IQR), *n* = 5
1–1	88.5 (77–99)	75 (63–80)	84 (79–89.5)
1–2	75 (75–76)	74 (47–79.5)	87 (67–90)
1–3	67.5 (63–75)	50 (30–67.5)	N/A
1–4	76 (75–80)	50 (39.5–74.5)	65 (57–82.5)
1–5	76 (75–80)	48 (39.5–62.5)	70 (54–77.5)
2–1	75 (75–75)	54 (37.5–67.5)	65 (47–75)
2–2	75 (75–75)	50 (44.5–67.5)	67 (51.5–75)
2–3	75 (75–82)	60 (42.5–77)	80 (52–90)
2–4	75 (75–82)	63 (45.5–72)	80 (53.5–91)
2–5	75 (75–82)	70 (40–83)	82 (59–96.5)
2–6	75 (75–82)	80 (44–90)	90 (58–95.5)
2–7	75 (75–75)	50 (35–65)	66 (38.5–75.5)

Abbreviations: IQR, interquartile range; N/A, not applicable.

## 4 DISCUSSION

In the current postgraduate clinical environment, there is variability in pediatric GI trainees' ability to attain autonomy by the completion of fellowship. Further, as new ACGME requirements for general pediatrics residency training move to encompass more outpatient care starting in July 2025, the expertise and confidence of incoming fellows in dealing with high acuity, subspecialty inpatients may further degrade.^19^ Medical education innovations provide alternate methods to help trainees achieve expertise. Interventions like simulation can supplement standard clinical training, move trainees more quickly through expected milestones, and launch independent physicians, which will directly benefit patient safety, clinical efficiency, and even the economics of training and clinical care. With rare high‐morbidity and mortality events in subspecialty pediatric care, simulation curricula could have an even greater clinical safety impact.

At Boston Children's Hospital, despite being a high‐volume, quaternary referral center, a large percentage of current GI fellows and recent graduates reported that they had not meaningfully cared for patients with complex liver disease. Among those fellows who did identify having cared for this patient population, they overwhelmingly felt that they could not confidently lead complex discussions or make independent clinical decisions, and cited lack of expertise as the biggest factor impeding autonomy. The feeling of decreased expertise may be due to the relatively much higher volume, daily repetition, and overall exposure that pediatric GI fellows experience with core GI disease processes as compared to hepatology. To our knowledge, an educational curriculum using simulation as a novel educational tool to help fill this gap in trainee clinical knowledge in pediatric hepatology has not been reported.

As such, this simulation curriculum, aimed to train fellows in sentinel events of pediatric hepatology, is the first to be implemented in a pediatric GI fellowship. Determinants of success in feasibility included smooth communication between the BCH Immersive Design Systems group and our team, commitment from the pediatric hepatology multidisciplinary team, who acted as facilitators, as well as from fellowship leadership.

The in‐person simulation curriculum was immediately impactful for its target fellow learners, who cited the collaborative, psychologically safe learning environment as a major key to success. We found that the ability for near‐peer teams to work together through complex, high‐stakes clinical decision making decreased the cognitive load of the experience and helped situate the learners optimally on the Yerkes–Dodson Law bell curve—that is, in a learning environment where participants are not so anxious as to inhibit performance but engaged enough to fully participate.^20^ Senior fellows were chosen to participate for both practical reasons (more flexibility in scheduling) as well as educational reasons, given they are closer to independent practice and may have higher engagement as they anticipate their first attending positions. Additionally, first‐year fellows have such a heavy clinical load that we felt they may not have the ability to digest and reflect on the experience adequately.

The online spaced learning activity provided another unanticipated boost in confidence levels across most learning objectives, despite having no additional didactics interwoven in the cases. This finding is supported by the theory of “retrieval practice,” a strategy whereby bringing information to mind via low‐stakes testing enhances and boosts durable learning.^21^ This supports the benefit of ongoing simulation opportunities (both in person and online) to those who do not have the opportunity for adequate retrieval practice from classical clinical training. There may also be a robust increase in learner confidence because of the durability of knowledge gained during the initial simulation experience.

The primary limitations of this curriculum pilot are its implementation in a single fellowship training program as well as dependence on self‐reporting surveys of confidence level, lack of clear measure of medical knowledge, and no control group of fellow trainees. While this pilot was targeted at a single fellowship class at one training program, because BCH has large class cohorts, this allows for a larger number of fellows with similar, standard training experiences to participate in the pilot. In turn, this provides a better baseline assessment of the impact on a larger number of fellows.

Another limitation is the pilot curriculum's use of the BCH simulation center and experienced staff, including technicians, coordinators, and educators. Not all programs have access to a large, well‐resourced simulation center, which may make the generalizability to other smaller programs more challenging. A future direction includes using the current case design and translating the experience to a simulation with lower physical fidelity (e.g., in a conference room) while still maintaining high psychological fidelity. General participant feedback supports this potential modification but would need to be formally assessed.

## 5 CONCLUSION

This is the first simulation curriculum designed for pediatric GI trainees. This innovation—the creation of a simulation experience for high morbidity and mortality events in a medical subspecialty—has not been previously reported. With the proven feasibility of the pilot, we anticipate that this experience could help expand and standardize the training of pediatric GI fellowship training across the country, regardless of fellowship program size or transplant center designation, and thereby impact the care of children with liver disease.

## CONFLICT OF INTEREST STATEMENT

The authors declare no conflicts of interest.
